# “Kill the Enemy”: Can Violence be Learned in Children by Activating Mirror Neurons Through Video Games?

**DOI:** 10.2174/0117450179408826250807093502

**Published:** 2025-08-11

**Authors:** Mauro G. Carta, Elisa Cantone, Fatma Charfi

**Affiliations:** 1 University of Cagliari, Cagliari, Italy; 2 University Tunis el Manar, Tunis, Tunisia

## Abstract

The impact of Violent Video Games (VVGs) on childhood development remains a subject of debate. While virtual reality has shown promise in enhancing social cognition through mirror neuron activation, concerns persist regarding the potential for video games to foster learned aggression, particularly in the absence of empathic or punitive feedback. Evidence regarding short-term desensitization effects is mixed, and long-term studies are scarce. Importantly, most existing research focuses on individuals exposed to video games after the age of eight, overlooking critical developmental periods marked by heightened neural plasticity. Early childhood exposure to violent content may be influenced by insecure attachment patterns, and this interaction may have consequences in socio-emotional learning. Factors, such as hyperactivity and parental absence, may further correlate with these effects. Despite these concerns, the presence of engaged caregivers has been shown to mitigate potential harm. There is an urgent need for longitudinal research and policies that promote responsible, adult-mediated video game use in early childhood.

## 1 LEARNING BY VIRTUAL REALITY AND IMPLICATIONS FOR VIDEO GAMING

A growing body of research has shown that cognitive remediation exercises conducted through virtual reality may enhance social intelligence in individuals with bipolar disorder [1], ADHD [2], and autism spectrum disorder [3]. It has therefore been hypothesized that the stimulation of mirror neurons induced by virtual reality, and the consequent learning through imitation, could, in this context, be even more intense than what occurs in real-life situations [4]. This could explain the remarkable outcomes observed with this technique [5].

By analogy, it could be speculated that conflict-based video games, often referred to as violent video games (VVGs), such as those involving rewards for “killing the enemy”, might reinforce the acquisition of violent responses and subsequent aggressive behaviors. This may be due to the lack of either empathic or punitive feedback that typically follows a violent act. For instance, a child who performs an aggressive gesture against an opponent during a sporting event may experience negative reinforcement through observing the pain caused or by facing reactions from opponents and sanctions from referees or coaches. In contrast, when a child “kills” an enemy in a video game, only positive reinforcement is provided, with no negative consequences or learning regarding the inflicted suffering.

## 2 MIXED EVIDENCE ON VIOLENT VIDEO GAMES AND AGGRESSION

To date, studies on the desensitization effects, namely the diminished emotional responses and the possible short-term induction of violent behavior from violent video games, have not yielded unequivocal and conclusive results. Some attempts have been made to demonstrate short-term desensitization effects using physiological reactivity parameters in response to violent stimuli, such as variations in heart rate response (*e.g*., lack of acceleration) or the absence of skin conductance responses [6-8]. A reduction in the P300 component in response to violent images after VVG exposure has also been demonstrated [9, 10]. However, other studies comparing responses to violent versus non-violent versions of the same game did not identify significant differences in skin conductance or heart rate responses to appropriate stimuli [11, 12]. A 2010 meta-analysis concluded that VVG exposure was associated only with short-term decreases in empathy and increased desensitization [13]. Conversely, a more recent study employing functional magnetic resonance imaging (fMRI) investigated whether relatively short-term exposure to violent video games in non-habitual male players (of unspecified young age) could reduce emotional empathy and increase violent responses in real life. However, it found no significant evidence of reduced empathic or emotional responses to violence compared to controls who had played non-violent games [14].

At the time of the aforementioned meta-analysis [13], the lack of long-term studies was already evident, and such studies have remained scarce. One study suggested possible empathy reduction and desensitization effects [15], while others did not confirm these findings [16, 17].

## 3 LIMITATIONS IN CURRENT RESEARCH: THE RELEVANCE OF EARLY LEARNING

A common feature of the more impactful studies is that they selected participants who began playing video games after the age of 8 [17] or young individuals who had never played video games [14].

Research conducted in various countries and cultural contexts has shown that many children today begin playing video games before the age of two. For example, converging results have been reported from studies in the USA [18], Turkey [19], and Italy [20, 21].

Certain behaviors and core competencies are primarily acquired during early childhood, a period when the brain is highly plastic, meaning it is significantly more flexible and receptive than in adulthood. These include well-known functions such as language acquisition [22] and basic visuo-perceptual abilities [23]. Understanding how modern approaches, such as machine learning, can predict developmental outcomes highlights the critical importance of this period for early interventions [24]. Similarly, attachment patterns, *i.e*., how a child emotionally relates to others, develop during the early years of life [25, 26]. Early experiences of neglect or abuse can impair the ability to form secure attachments in adulthood [27]. Research has indicated that attachment insecurity can mediate the relationship between childhood maltreatment and violent behavior in adulthood. Specifically, insecure attachment has been associated with a greater tendency toward violent behavior, suggesting that early relational difficulties can negatively impact future social behavior and aggressive responses [28, 29].

More broadly, these findings imply that complex patterns of violent response can be significantly influenced by conditioning during the earliest years of life.

This is precisely the developmental period during which children’s first encounters with violent content, such as in video games, are becoming more frequent. While it is unlikely that a two-year-old would encounter violent games, it is highly probable by the age of four. At this stage, the attachment system is still developing, and socially reinforced responses can leave enduring imprints that may shape lifelong behavioral patterns.

## 4 POTENTIAL MODERATING AND INTERACTING FACTORS

The influence of video games on aggressive behavior is undoubtedly complex and interacts with many other factors. One important aspect involves certain characteristics, such as hyperactivity and novelty seeking, traits that are not pathological in themselves but can contribute to a child’s tendency to avoid parental presence. These traits are often linked to a vulnerability to rhythm dysregulation, which may represent a risk factor. This is particularly relevant given that the light pollution resulting from excessive time spent on video games can affect melatonin production and stress hormone levels [30].

A major limitation of current knowledge is that much of it is derived from studies involving neurodivergent or non-neurotypical children. However, several studies suggest that the attentive and conscious presence of parents, particularly during early childhood [31], can mitigate the negative effects of video games. The active involvement of adults can transform video gaming into an educational and developmental opportunity [32]. This highlights the importance of the context of use and the family environment in shaping the impact of video games on children.

## 5 LIMITATIONS

While this work offers thoughtful insights into the topic, it is important to acknowledge its limitations in the scientific approach employed, which may impact the analysis of various scientific perspectives.

## CONCLUSION

Recent studies [33] have demonstrated that it is possible to detect brain responses associated with social behavior in real-time by simulating complex situations involving play and deception. Tools similar to functional near-infrared spectroscopy (fNIRS) could be useful for studying the impact of violent video games on the brain functions of children and their neural network activation. Given the increasing exposure of children to video games, it is essential to promote longitudinal studies to better understand their long-term effects. Combining interventions on parenting skills in these areas will enable research to provide a basis for the development of informed guidelines and educational policies.

## AUTHORS’ CONTRIBUTIONS

It is hereby acknowledged that all authors have accepted responsibility for the manuscript's content and consented to its submission. They have meticulously reviewed all results and unanimously approved the final version of the manuscript.

## LIST OF ABBREVIATIONS

VVGs: Violent Video Games
ADHD: Attention Deficit Hyperactivity Disorder
fMRI: Functional Magnetic Resonance Imaging
fNIRS: Functional Near-infrared Spectroscopy
